# Crop Row Detection in Maize Fields Inspired on the Human Visual Perception

**DOI:** 10.1100/2012/484390

**Published:** 2012-04-30

**Authors:** J. Romeo, G. Pajares, M. Montalvo, J. M. Guerrero, M. Guijarro, A. Ribeiro

**Affiliations:** ^1^Department of Software Engineering and Artificial Intelligence, Faculty of Informatics, University Complutense, 28040 Madrid, Spain; ^2^Department of Computer Architecture and Automatic, Faculty of Informatics, University Complutense, 28040 Madrid, Spain; ^3^Artificial Perception Group, Center for Automation and Robotics (CAR), CSIC-UPM, 28500, Arganda del Rey, Madrid, Spain

## Abstract

This paper proposes a new method, oriented to image real-time processing, for identifying crop rows in maize fields in the images. The vision system is designed to be installed onboard a mobile agricultural vehicle, that is, submitted to gyros, vibrations, and undesired movements. The images are captured under image perspective, being affected by the above undesired effects. The image processing consists of two main processes: image segmentation and crop row detection. The first one applies a threshold to separate green plants or pixels (crops and weeds) from the rest (soil, stones, and others). It is based on a fuzzy clustering process, which allows obtaining the threshold to be applied during the normal operation process. The crop row detection applies a method based on image perspective projection that searches for maximum accumulation of segmented green pixels along straight alignments. They determine the expected crop lines in the images. The method is robust enough to work under the above-mentioned undesired effects. It is favorably compared against the well-tested Hough transformation for line detection.

## 1. Introduction

### 1.1. Problem Statement

The increasing development of robotics equipped with machine vision sensors applied to Precision Agriculture is demanding solutions for several problems. The robot navigates and acts over a site-specific area of a larger farm [1], where one important part of the information is supplied by the vision system.

An important issue related with the application of machine vision methods is that concerning the crop row and weed detection, which has attracted numerous studies in this area [2–6]. This will allow site-specific treatments trying to eliminate weeds and to favor the growth of crops.

The robot navigates on a real terrain presenting irregularities and roughness. This produces vibrations and also swinging in the pitch, yaw, and roll angles. Moreover, the spacing of crop rows is also known. Because of the above, the crop rows are not projected on the expected locations in the image. On the other hand the discrimination of crops and weeds in the image is a very difficult task because their red, green, and blue spectral components display similar values. This means that no distinction is possible between crops and weeds based on the spectral components. Thus the problem is to locate the crop rows in the image. To achieve this goal, in this paper we propose a new strategy that exploits the specific arrangement of crops (maize) in the field and also applies the knowledge of perspective projection based on the camera intrinsic and extrinsic parameters. This method is inspired by the human visual perception and like humans applies a similar reasoning for locating crop rows in the images, although it exploits the camera system geometry, because it is available. As we will see in the next section, the crop row location is not new and has been considered already in the literature; the method proposed in this paper gains advantage over existing approaches because it has been designed to achieve high effectiveness in real-time applications. This makes the main contribution of this paper. The method does not include a segmentation step, which is found in most other methods for plant detection. This tries to avoid time consumption as compared to other strategies. The segmentation step has been replaced by a simple thresholding method, where the threshold is previously established by applying a learning-based fuzzy clustering strategy. Cluster centers for green textures are obtained during an off-line learning phase, and then this knowledge is exploited during the online decision phase. Crop row detection is an easy step where simple straight lines are traced, based on perspective projection, looking for specific pixels alignments defining crop rows.

Moreover it applies downsampling for reducing image sizes. All above steps are oriented to gain time reduction during the computational process. The proposed approach is favorably compared against some existing approaches in both effectiveness and time reducing.

### 1.2. Revision of Methods

Several strategies have been proposed for crop row detection. Fontaine and Crowe [7] tested the abilities of fourth-line detection algorithms to determine the position and the angle of the camera with respect to a set of artificial rows with and without simulated weeds. These were stripe analysis, blob analysis, linear regression, and Hough transform.

**(1) Methods Based on the Exploration of Horizontal Strips**

Søgaard and Olsen [8] apply RGB color image transformation to grayscale. This is done by first dividing the color image into its red, green, and blue spectral channels and then by applying the well*-*tested methods to extract living plant tissue described in [9]. After this, the greyscale image is divided into horizontal strips where maximum grey values indicate the presence of a candidate row, each maximum determines a row segment, and the center of gravity of the segment is marked at this strip position. Crop rows are identified by joining marked points through a similar method to the one utilized in the Hough transform or by applying linear regression. Sainz-Costa et al. [6] have developed a strategy based on analysis of video sequences for identifying crop rows. Crop rows persist along the directions defined by the perspective projection with respect the 3D scene in the field. Exploiting this fact, they apply greyscale transformation, and then the image is binarized by thresholding. Each image is divided into four horizontal strips. Rectangular patches are drawn over the binary image to identify patches of crops and rows. The gravity centers of these patches are used as the points defining the crop rows, and a line is adjusted considering these points. The first frame in the sequence is used as a lookup table that guides the full process for determining positions where the next patches in subsequent frames are to be identified. Hague et al. [10] transform the original RGB image to gray scale. The transformed image is then divided into eight horizontal bands. The intensity of the pixels across these bands exhibits a periodic variation, due to the parallel crop rows. Since the camera characteristics, pose and the crop row spacing are known a priori, the row spacing in image pixels can be calculated for each of the horizontal bands using a pinhole model of the camera optics. A bandpass filter can then be constructed which will enhance this pattern and has a given frequency domain response. Sometimes horizontal patterns are difficult to extract because crops and weeds form a unique patch.

**(2) Methods Based on the Hough Transformation**

According to Slaughter et al. [11], one of the most commonly used machine vision methods for identifying crop rows is based upon the Hough [12] transform. It was intended to deal with discontinues lines, where the crop stand is incomplete with gaps in crop rows due to poor germination or other factors that result in missing crop plants in the row. It has been intended for real-time automatic guidance of agricultural vehicles [13–16]. It is applied to binary images, which are obtained by applying similar technique to the ones explained above, that is, RGB image transformation to grayscale and binzarization [3, 4, 17]. Gée et al. [18] apply a double Hough transform under the assumption that crop rows are the only lines of the image converging to the vanishing point, the remainder lines are rejected, additional constraints such as interrow spacing and perspective geometry concepts help to identify the crop rows. It is required to determine the threshold required by the Hough transform to determine maximum peaks values [19, 20] or predominant peaks [21]. Depending on the crop densities, several lines could be feasible, and a posterior merging process is applied to lines with similar parameters [3, 4, 17]. Ji and Qi [22] report that Hough transform is slow due to the huge computation; they propose a randomized Hough transform to reduce computational time. Some modifications have been proposed to improve the Hough transformation such as the one proposed in Asif et al. [23], which apply the Hough only to those points which are edge points along the crops. But this requires the application of techniques for edge extraction. Also the randomized Hough transformation has been proposed with this goal [22]. It is intended to avoid redundant computations in the Hough transform. It operates iteratively by randomly sampling a set of points to compute a single localization in the Hough space. Since two pixels are trivially collinear, the parameters of the line on which they lie can be estimated. These parameters are used to increment the accumulator cell in the Hough space. In summary, the Hough transform is computationally expensive and the randomized Hough transform requires selecting pairs of points to be considered as a unique line, that is, pairs of points belonging to a crop row. If we apply this technique in images where edge points have been extracted, the selection of those pairs becomes more complex. Furthermore, the computational cost of Hough-based algorithms is very sensitive to the image resolution after down-sampling, but also when weeds are present and irregularly distributed, this our case, this is could cause the failure detection. Moreover, as the weed density increases the crop row detection becomes more and more difficult.

**(3) Vanishing Point Based**

Pla et al. [24] propose an approach which identifies regions (crops/weeds and soil) by applying color image segmentation. They use the skeleton of each defined region as a feature to work out the lines which define the crop. The resulting skeletons of each region can be used as curves which define the underlying structure of the crop and to extract the straight lines where the plants, and soil rows lie. Segments in the skeletons are defined as chains of connected contour points and they must be of a defined length. This allows selecting candidate lines for crop row detection, among all candidates the ones that meet the vanishing point. The vanishing point is detected using previous information about the vanishing point found in the previous images, performing a sort tracking on the vanishing point. This process is highly dependent on the skeletons, which are not always easy to extract and isolate, particularly considering that crops and weeds patches appear overlapped among them.

**(4) Methods Based on Linear Regression**

Some of the techniques above apply this approach. Billingsley and Schoenfisch [25] reported a crop detection system that is relatively insensitive to additional visual “noise” from weeds. They used linear regression in each of three crop row segments and a cost function analogous to the moment of the best-fit line to detect lines fitted to outliers (i.e., noise and weeds) as a means of identifying row guidance information. As mentioned above, Søgaard and Olsen [8] also apply linear regression, which is a feasible approach if weed density is low and pixels belonging to crop rows are well separated. Otherwise it is highly affected by pixels belonging to weeds because of their strong contribution to line estimation.

**(5) Stereo-Based Approach**

Kise et al. [26] and Kise and Zhang [27] developed a stereovision-based agricultural machinery crop row tracking navigation system. Stereoimage processing is used to determine 3D locations of the scene points of the objects of interest from the obtained stereoimage. Those 3D positions, determined by means of stereoimage disparity computation, provide the base information to create an elevation map which uses a 2D array with varying intensity to indicate the height of the crop. This approach requires crops with significant heights with respect the ground. Because in maize fields, during the treatment stage, the heights are not relevant, it becomes ineffective in our application. Rovira-Más et al. [28] have applied and extended stereovision techniques to other areas inside Precision Agriculture. Stereo-based methods are only feasible if crops or weeds in the 3D scene display a relevant height and the heights differ in both kind of plants.

**(6) Methods Based on Blob Analysis**

This method finds and characterizes regions of contiguous pixels of the same value in a binarized image [7]. The algorithm searches for white blobs (interrow spaces) of more than 200 pixels, as smaller blobs could represent noise in the crop rows. Once the blobs are identified, the algorithm determines the angle of their principal axes and the location of their center of gravity. For a perfectly straight white stripe, the center of gravity of the blob was over the centerline of the white stripe, and the angle was representative of the angle of the interrow spaces. The algorithm returned the angle and center of gravity of the blob closest to the centre of the image. Identification of blobs in images infested with weeds in maize fields becomes a very difficult task, because weeds and crops under overlapping in localized areas produce wide blobs.

**(7) Methods Based on the Accumulation of Green Plants**

Olsen [29] proposed a method based on the consideration that along the crop row an important accumulation of green parts in the image appears. The image is transformed to gray scale, where green parts appear clearer that the rest. A sum curve of gray levels is obtained for a given rectangular region exploring all columns in the rectangle. It is assumed that vertical lines follow this direction in the image. Images are free of perspective projection because they are acquired with the camera in orthogonal position. A sinusoidal curve is fitted by means of least squares to the sum curve previously obtained. Local maxima of the sinusoid provide row centers locations. This is a simple and suitable method, which can be still simplified but it is not of our interest because of the fact that the images we work with are taken from the tractor under perspective projection but not orthogonal. In this paper we exploit the idea of green plant accumulation under a simpler strategy.

**(8) Methods Based on Frequency Analysis**

Because crop rows are vertical in the 3D scene, they are mapped under perspective projection onto the image displaying some behavior in frequency domain. Vioix et al. [30] exploit this feature and apply a bidimensional Gabor filter, defined as a modulation of a Gaussian function by a cosine signal. The frequency parameter required by the Gabor filter is empirically deduced from the 2D-Fast Fourier Transform [31]. Bossu et al. [32] apply wavelets to discriminate crop rows based on the frequency analysis. They exploit the fact that crop rows are well localized in the frequency domain; thus selecting a mother wavelet function with this frequency the crop rows can be extracted. In maize fields where the experiments are carried out, crops do not display a clear frequency content in the Fourier space, therefore the application of filters based on the frequency becomes a difficult task.

### 1.3. Motivational Research and Design of the Proposed Strategy

Our work is focused on crop row detection in maize for specific treatments requiring discrimination among crops and weeds. This means that crop rows must be identified and located in the image conveniently. Some of the requirements proposed by Astrand [33] and reported in [11] for guidance systems can be considered for crop row detection; the problem is essentially similar. Therefore, our system is designed to be

- able to locate crop rows with the maximum accuracy as possible,
- able to work on real-time,
- able to work on sown crops, not manually planted, which means that weeds and crops grow simultaneously displaying, at the early growth stage of the treatment, similar heights and also similar spectral signatures. This means that discrimination between crops and weed cannot be made by height or spectral signatures only,
- able to work when plants are missing in the row,
- able to work when there is high weed pressure,
- able to work under different weather (luminance) conditions,
- able to locate crop rows with the least assumptions and constraints.

The aim of this study is to present a general method for identifying crop rows in maize fields from the images. We exploit the advantages of the existing methods introduced above, extracting the main ideas, and design a new strategy for crop row detection inspired on the human visual perception abilities which is able to work in real time.

This method is also dedicated to be applied in maize with crop row spacing and also to deal with and without seedling spacing. It is summarized in the two main steps as follows.

- Segmentation of green plants (crop and weeds).
- Crop row identification.

## 2. Materials and Methods

### 2.1. Images

The images used for this study correspond to maize crops. They were captured with a Canon EOS 400D camera during April/May 2011 in a 1.7 ha experimental field of maize in La Poveda Research Station, Arganda del Rey, Madrid. All acquisitions were spaced by five/six days, that is, they were obtained under different conditions of illumination and different growth stages. The digital images were captured under perspective projection and stored as 24-bit color images with resolutions of 5 MP saved in RGB (red, green, and blue) color space in the JPG format. The images were processed with MATLAB R2009*a* [34] under Windows 7 and Intel Core 2 Duo CPU, 2.4 GHz, 2.87 GB RAM. A set of 350 images were available for processing.

**Figure 1:**
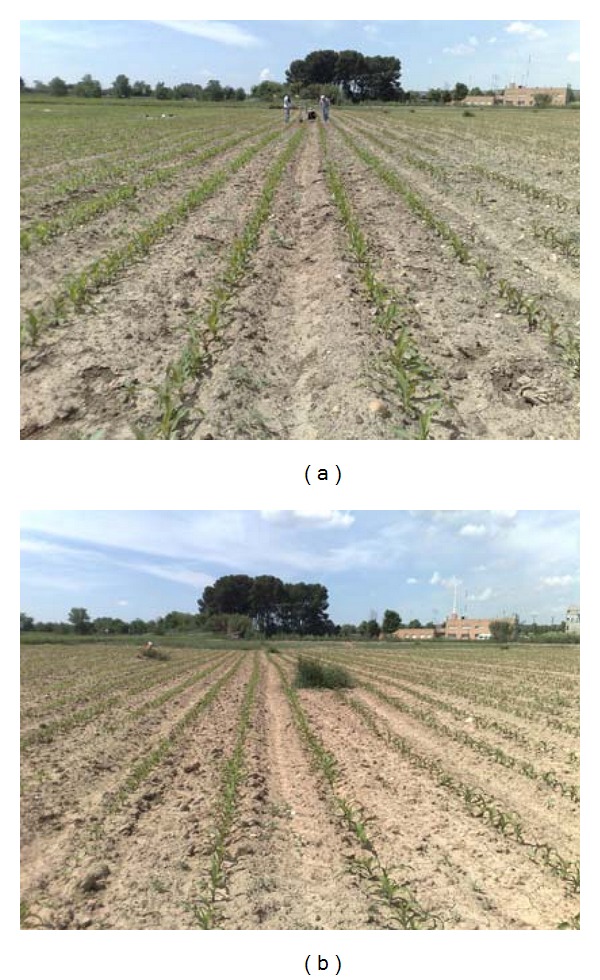
Different brightness due to different weather conditions: (a) darker; (b) clearer.

**Figure 2:**
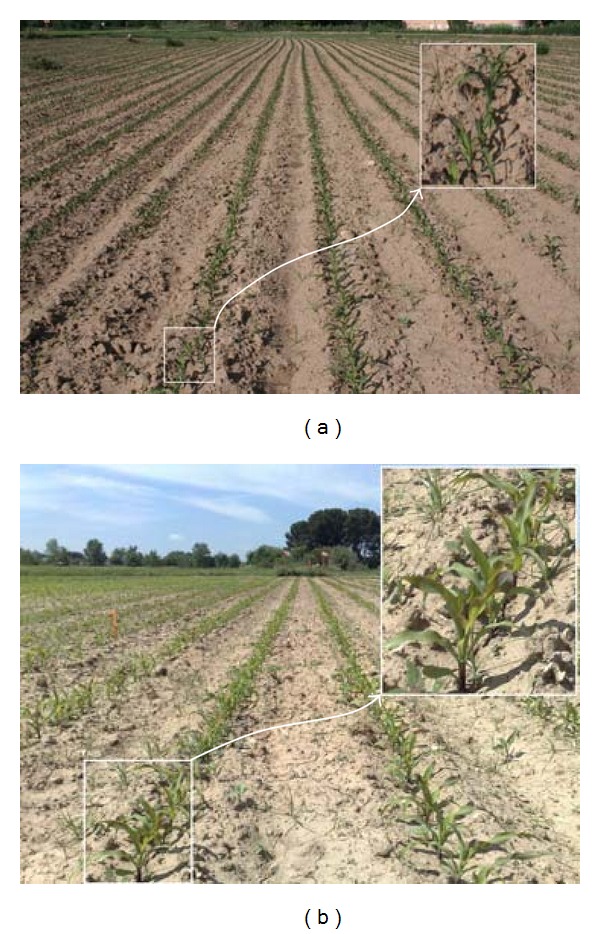
Different crop growth stages: (a) low; (b) high.

**Figure 3:**
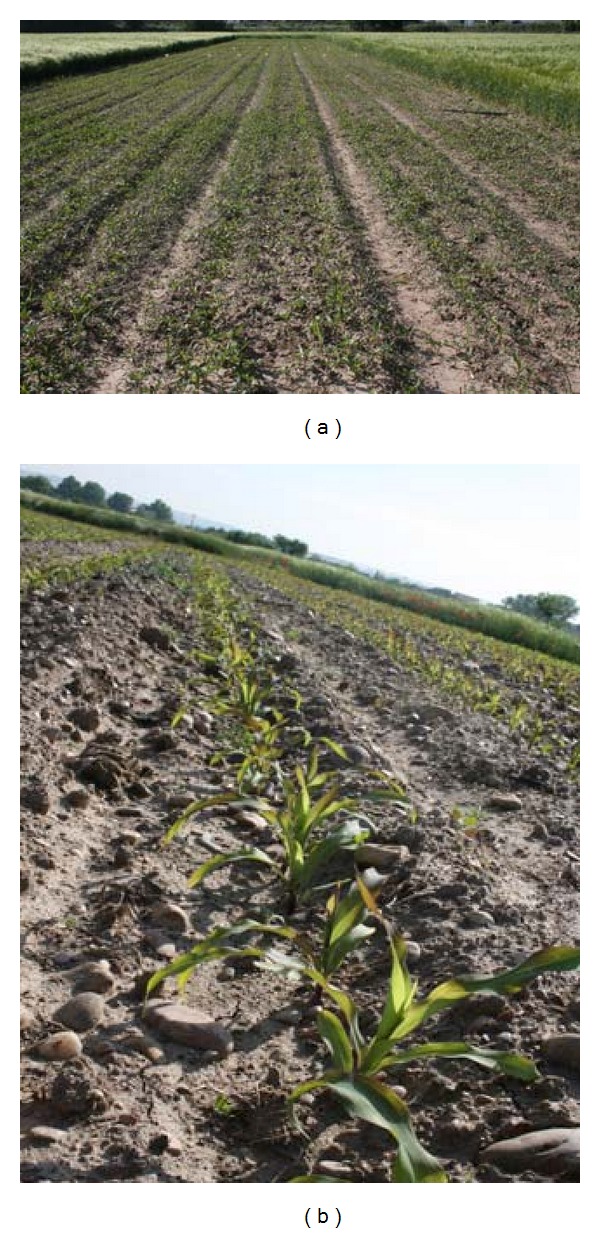
Different yaw, pitch and roll angles, and heights from the ground.

**Figure 4:**
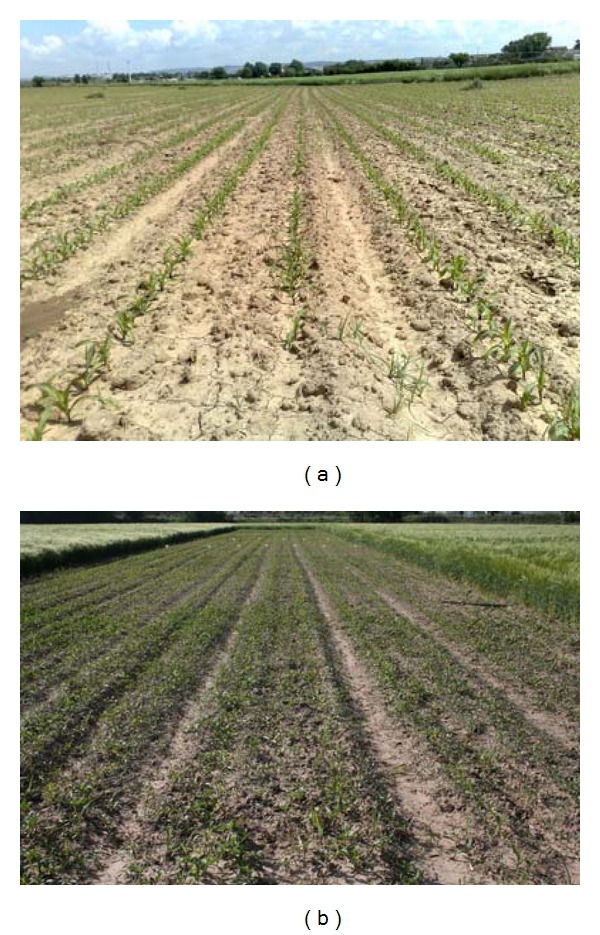
Different weed densities: (a) low; (b) high.

With the aim of testing the robustness and performance of the proposed approach, we have worked with images captured under different conditions, including different number of crop rows; these conditions have been identified in the real fields as possible and also those that could cause problems during the detection process in normal operation. The following is a list of representative images from the set of available images, illustrating some of such conditions:

- different brightness due to different weather conditions, Figures 1(a) and 1(b);
- different growth stages, Figures 2(a) and 2(b);
- different camera orientations, that is, different yaw, pitch and roll angles, and heights from the ground, Figures 3(a) and 3(b);
- different weed densities, Figures 4(a) and 4(b).

### 2.2. Image Segmentation: Green Plants Identification

For real-time applications is of great relevance to simplify this process as much as possible. Instead of using vegetation indices [9, 35], which require an image transformation from RGB color space to gray scale, we used a learning-based approach with the goal of obtaining the percentage of the green spectral component with respect to the remainder, which allowed us to consider a pixel belonging to a green plant. This relative percentage is intended to deal with illumination variability so that it determines relative values among the three spectral RGB components that identify green plants. This is carried out by applying a *fuzzy clustering* approach. Under this approach there is a learning phase which is applied to during offline activity for computing the relative percentage or threshold and a decision phase where the threshold is applied without additional computation.

 The learning phase was designed as follows. From the set of available images we randomly extracted *n* training samples, stored in *X*, that is, *X* = {**x**_1_, **x**_2_,…, **x**_*n*_} ∈ *ℜ* ^*d*^, where *d* is the data dimensionality. Each sample vector **x**_*i*_ represents an image pixel, where its components are the three RGB spectral components of that pixel at the original image location (*x*, *y*). This means that in our experiments the data dimensionality is *d* = 3. Each sample is to be assigned to a given cluster *w*_*j*_, where the number of possible clusters is *c*, that is, *j* = 1,2,…, *c*. In the proposed approach *c* is set to 2 because we were only interested in two types of textures, that is, green plants (crop/weeds) and the remainder (soil, debris, stones).

 The samples in *X* are to be classified based on the well-known fuzzy clustering approach that receives the input training samples **x**_*i*_ and establishes a partition, assuming the number of clusters *c* is known. The process computes for each **x**_*i*_ at the iteration *t*, its degree of membership in the cluster *w*_*j*_(*μ*_*i*_^*j*^) and updates the cluster centers **v**_*j*_ as follows [36]:

(1) $$\begin{array}{c} \mu_{i}^{j}\left(t+1\right)=\frac{1}{\sum_{r=1}^{c}{\left(d_{ij}\left(t\right)/d_{ir}\left(t\right)\right)}^{2/\left(b-1\right)}};\\ v_{j}\left(t+1\right)=\frac{\sum_{i=1}^{n}{\left[\mu_{i}^{j}\left(t\right)\right]}^{b}x_{i}}{\sum_{i=1}^{n}{\left[\mu_{i}^{j}\left(t\right)\right]}^{b}}. \end{array}$$

*d*_*ij*_^2^ ≡ *d* ^2^(**x**_*i*_, **v**_*j*_) is the squared Euclidean distance. The number *b* is called the exponential weight [37, 38], *b* > 1. The stopping criterion of the iteration process is achieved when ||*μ*_*i*_^*j*^(*t* + 1) − *μ*_*i*_^*j*^(*t*)|| < *ε*  for  all  *ij* or a number *t*_max⁡_  of iterations is reached.

The method requires the initialization of the cluster centers, so that (1) can be applied at the iteration *t* = 1. For this purpose, we applied the pseudorandom procedure described in Balasko et al. [39].

1. Perform a linear transform *Y* = *f*(*X*) of the training sample values so that they range in the interval [0,1].
2. Initialize $v=2D\overset{®}{M}\circ R+D\overset{®}{m}$, where $\overset{®}{m}$ is the mean vector for the transformed training samples values in *Y* and $\overset{®}{M}=\max\left(\text{abs}\left(Y-\overset{®}{m}\right)\right)$, both of size 1 × *d*; *D* = [1 …. 1]^*T*^ with size *c* × 1; **R** is a *c* × *d* matrix of random numbers in [0,1]; the operation ∘ denotes the element by element multiplication.

Once the learning process is finished we obtain two cluster centers **v**_1_ and **v**_2_ associated to clusters *w*_1_ and *w*_2_. Without loss of generality, let **v**_1_ ≡ {*v*_1*R*_, *v*_1*G*_, *v*_1*B*_} the one associated to the green plants. It is a 3-dimensional vector where its components *v*_1*R*_, *v*_1*G*_, and *v*_1*B*_ represent the averaged values for the corresponding RGB spectral components; thus the threshold value for discriminating among green plants and the remainder ones is finally set to *T*_*G*_ = *v*_1*G*_/(*v*_1*R*_ + *v*_1*G*_ + *v*_1*B*_).

Once *T*_*G*_ is available, the green parts on the images are identified assuming the corresponding RGB pixels contain the G spectral value greater than *T*_*G*_. Therefore, during the online identification process only is required the logical comparison.

### 2.3. Crop Row Identification

Once green parts were extracted in the image, next step was crop row identification. For such purpose we make use of the following constraints, based on the image perspective projection and the general knowledge about the maize field.

- The number of crop lines (*L*) to be detected is known and also the approximate *x* position or image column at the bottom of the image where every crop line starts. This assumption is based on the system geometry and the image perspective projection.
- We are going to detect crop lines that start from the bottom of the image and end at the top of the image. Lines starting from both left and right sides of the image and vanishing at the top are rejected. This is because image geometry allows to consider this situation. An extension of this algorithm could be done to detect those lines with its corresponding computing time cost.
- All the images have been acquired with a camera onboard a tractor and pointing in the same direction as the crop lines, therefore images are mapped under in perspective projection and the crop lines converge in the well-known vanishing point. This constraint is inspired by methods based on the vanishing point, as described in the introduction. Though crop lines are parallel, distance between crop lines seems to be greater at the bottom of the image than at the top, due to perspective. This algorithm works considering that crop lines are going to have that appearance in the image with a range of tolerance that can be adjusted depending on the stability of the tractor and the evenness of the ground. We assume that crop lines starting from the left bottom of the image have a clockwise slope and lines starting from the right bottom of the image have an anticlockwise slope. The bigger the range of tolerance the higher the computing time. In this paper we have used a 15% of tolerance which means that crop lines may vary from one image to the next one a 15% of the width of the image.

The algorithm works as follows.

**Figure 5:**
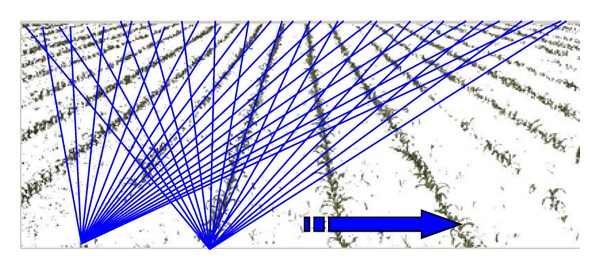
Lines traced from every pixel of the bottom row in the image.

**Figure 6:**
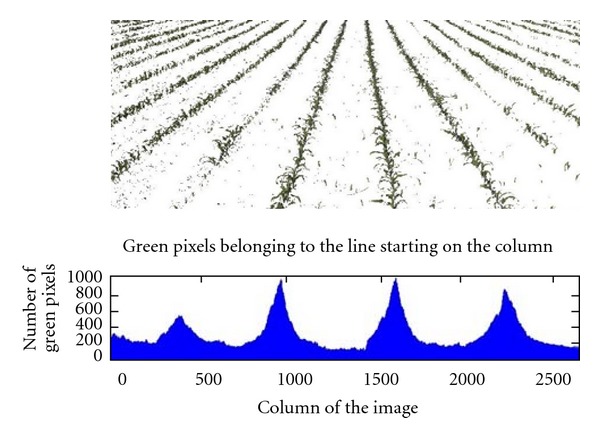
Number of green pixels found for the best line of every pixel of the bottom line.

1. From every pixel in the bottom row we trace all the possible lines starting on that pixel and ending on every pixel of the top line, that is, if the image has *N*-columns, we will trace *N* ^2^ lines from every pixel of the bottom row, which means that we finally trace *N* ^2^ lines. This number of calculated lines is the highest number of lines in case we make no constraints. Nevertheless, as we will see in step 4 and 5 important constrains can be applied to reduce this number.  Figure 5 shows the bean of lines traced for two pixels placed at the bottom row of the image. For illustrating purposes we have traced broad beams of lines, but the number of lines to cope with all possible situations, but this number could be considerably reduced by applying previous knowledge, like the slope. This is applied in this work as described below, reducing the computational cost.
2. For every traced line starting on a pixel of the bottom row, we count the number of “green” pixels that belong to that line. This is possible because the image has been already segmented and pixels belonging to green parts have been identified. Now, the line with the highest number of green pixels is the candidate line to represent the crop row for that pixel of the bottom row.
3. We repeat the same procedure for all the pixels of the bottom row, and finally we obtain c-candidates lines, that is, one for every pixel of the bottom row. As we can see in Figure 6, every pixel location in the bottom row of the image has a value for its best line. Those values become higher as the represented line approaches the real crop line. They are the peaks in the lower part in Figure 6.
4. Since we know the number of crop lines to be detected and also where they roughly start at the bottom row, we can choose the closer and highest values, which are identified by peaks in the accumulator. With such a figure, assume the number of crop rows to be detected is four; so we look for four peaks that are conveniently spaced because of the crop rows arrangement in maize fields and also based on camera system geometry. This idea is inspired in methods based on the accumulation of green plants, described in the introduction.

The following are three considerations that can be applied to speed up the computational process from the point of view of a real-time application.

1. For each selected line we store the start and end points, obtaining the corresponding equation for the straight line.
2. Because of the perspective of the image it is not necessary to trace all the lines to the top row (as mentioned in step 1) but only those whose slope is according to what we expect. That is, if we are dealing with left pixels of the image we would trace only lines with a slope clockwise and without reaching the end of the right side of the image. For right pixels we would search for anticlockwise slopes starting from the right side and without reaching the left side of the image. This idea is based on the vanishing point concept, applied in some approaches as described in the introduction.
3. In addition to it, it is not necessary to trace lines pixel by pixel. Depending on the image resolution a “pixel step” can be used without affecting final result and reducing considerably the computational cost.
4. Notice that there are some values to be adjusted before the algorithm runs. These values depend on the images we are dealing with and on the stability of the camera. The higher the image resolution the higher the “pixel step” for lines calculation. Furthermore, the higher the stability of the tractor the thinner the range of pixels of the top row for tracing lines.

## 3. Results

Our proposed crop row detection (CRD) method consists on a first stage or learning phase where the threshold *T*_*G*_ is obtained for segmenting green plants. With such purpose we have processed 200 images, selected from the set of 350 images available, from which we have randomly extracted 40.000 training samples. The selected images cover the broad range of situations, that is, different number of rows, weather conditions, weed concentrations and growth states according to Figures 1 to 4.

With these training samples, we apply the fuzzy clustering procedure described in Section 2.2, from which we compute two cluster centers identifying both green plants (**v**_1_) and soil or other components (**v**_2_). Our interest is only focused on segmenting green plants, therefore, from **v**_1_ we compute the threshold *T*_*G*_ defined in Section 2.2 as the percentage of the green component in **v**_1_, that is, *T*_*G*_ = 0.37. This is the threshold finally used for image segmentation.

Table 1 displays both cluster centers **v**_1_ and **v**_2_ and the percentage of the highest value in the spectral components associated to each cluster center. As we can see green and soil pixels can be identified by the corresponding percentages, each one applied over the green and red spectral components. This was the general behavior observed for the set of images analyzed.

As mentioned during the introduction, the Hough transform has been applied in several methods for crop row detection, hence we compare the performance of our CRD approach against the Hough (HOU) transform. We have applied identical conditions to the Hough transform than the ones applied in our CRD approach, so it works in terms of comparability; they are synthesized as follows.

- Search for lines arising from the bottom of the image and ending at the top, that is, suspicious useless lines are not explored.
- Only are allowed lines with slopes close to the ones expected at each side of the image. Horizontal lines and many others that do not meet the above are rejected.
- The Hough transform is implemented to work under the normal representation, polar coordinates [40], with unit increments in the parameter representing the angle.

The comparison is established in terms of effectiveness and processing times. The effectiveness is measured based on the expert human criterion, where a line, which has been detected, is considered as correct if it overlaps with the real crop row alignment. Over the set of 350 images analyzed, we compute the average percentage of coincidences for both CRD and HOU. Also, because the main goal of the proposed approach is its profit for real time applications, we measure computational times. Also, with the goal of real-time, we have tested these performances for different image resolutions. As we can see image resolutions differ from the ones in the original images, these resolutions have been obtained by applying a down-sampling process to the original image.

This is intended under the idea that it is possible to reduce the image dimension retaining the main information without affecting the effectiveness and reducing the processing time.  Table 2 displays the results. The first column contain different image resolutions, which are obtained by selecting large regions of interest in each image, with horizontal and vertical sizes of 1940 × 2590 pixels, and these regions contains different number of crop rows under different configurations provided by the images displayed in Figures 1 to 4. These large regions are split by 10, 8, 6, and 4, which are, respectively the ones represented in Table 2. We have chosen this set of values because with them we obtain similar performances in terms of effectiveness with acceptable processing times. The effectiveness for higher resolutions is similar, but the processing times increase considerably. Below the lower resolution, the effectiveness decreases considerably.

**Figure 7:**
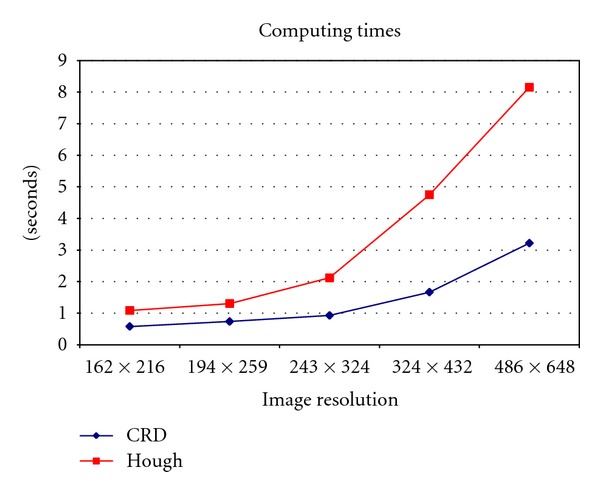
Times in seconds against the different image resolutions.

The second and third columns contain the percentage of effectiveness and columns fourth and fifth the processing times measured in seconds. All these measurements represent average values over the set of 350 images processed. For clarity, Figure 7 represents the processing times in Table 2, for the four resolutions studied.

From results in Table 2 we can infer that CRD outperforms HOU in terms of effectiveness, with a near constant value regardless the image resolution. With lower resolutions, that is, with image divisions above 12, this percentage decreases drastically, achieving values below 85%. This is because for low resolutions some important information in the images is lost. Thus, from values in Table 2 and because the processing time is lower with small image resolutions, from a real-time point of view, a suitable resolution with acceptable performance is the lowest, that is, the one for 162 × 216. From Figure 7, one can see that the increasing of time is not linear. For resolutions above 243×324 time differences are more pronounced.

The worst performance obtained for HOU can be explained because crops and weeds concentration produces a high density of values, representing peaks, in the cell accumulator. These values do not display a high clear value, theoretically representing a unique crop line. Moreover, the absolute maximum value around the expected crop line most times does not represent the correct line. Thus, it is necessary to define a patch selecting different high peak values for each expected line, which are averaged, to obtain the final value. Because this patch has not clear limits, its selection becomes a difficult task and errors in the selection produce errors in the crop row localization, which explain the worst performance of HOU against CRD.

## 4. Conclusions

We developed a new method for crop row detection that improves Hough-based methods in terms of effectiveness and computing time. The goal is its application to real-time implementations.

Furthermore, our approach has been proved to be robust enough to different images typologies.

The proposed method is robust enough to work in images under perspective projection. It can detect any number of crop lines with any slope converging in a vanishing point. It works with either high or low image resolutions.

Future works must be oriented toward weeds detection by establishing cells around crop lines and calculating the percentage of greenness of every cell. This should be intended for posterior actuations to kill weeds.

## Figures and Tables

**Table 1:** Percentage of the green spectral component for green plants and for other components (soil, debris, stones).

	Spectral component values	Percentage of the highest spectral component
**v**_1_ (green plants)	{137.80 140.68 106.07}	0.37 (green)
**v**_2_ (soil and other components)	{188.49 177.71 153.53}	0.36 (red)

**Table 2:** Performances of HOU and CRD approaches measured in terms of percentage of effectiveness and processing times.

*Image resolution (pixels)*	Percentage of effectiveness		Processing time (seconds)	
	HOU	CRD	HOU	CRD
162 × 216	86.3	97.1	1,088	0,580
194 × 259	89.4	97.3	1,305	0,737
243 × 324	89.1	97.3	2,120	0,928
324 × 432	90.9	97.4	4,752	1,667
486 × 648	91.1	97.5	8,153	3,216
